# An Unusual and Rare Metachronous Ipsilateral Ureteric Stump Metastasis Post Radical Nephrectomy

**DOI:** 10.7759/cureus.17727

**Published:** 2021-09-05

**Authors:** Ramandeep Chalokia, Chinedum Anosike, Lee Robinson, Catherine Manson, Manal Kumar

**Affiliations:** 1 Urology, Warrington and Halton Teaching Hospitals National Health Service Foundation Trust, Warrington, GBR; 2 Radiology, Warrington and Halton Teaching Hospitals National Health Service Foundation Trust, Warrington, GBR; 3 Pathology, Warrington and Halton Teaching Hospitals National Health Service Foundation Trust, Warrington, GBR; 4 Urology, Arrowe Park Hospital, Wirral University Teaching Hospital National Health Service Foundation Trust, Birkenhead, GBR

**Keywords:** computed tomography, chromophobe renal cell carcinoma, hematuria, ureteric stump metastases, metachronous

## Abstract

We report a case of recurrence of chromophobe renal cell cancer in the ipsilateral ureteric stump eight years later after the primary tumor was excised successfully. Before this detection of the recurrence, the patient had presented with recurrent episodes of hematuria four years after the radical nephrectomy was performed and the investigations were inconclusive. Eventually, the lesion was detected on flexible cystoscopy in the area of the right ureteric orifice protruding in the bladder. Transurethral resection of the tumor surprisingly revealed a chromophobe renal cancer with similar features seen in the primary tumor specimen. The patient underwent robotic-assisted laparoscopic excision of the ureteric stump with a cuff of the bladder and has been recurrence-free for five years on regular surveillance scans.

## Introduction

Renal cell cancer (RCC) is the most common neoplasm affecting the kidney and accounts for 2-3% of all cancers [1]. It exhibits varied manifestations and is also known to have unpredictable biological attributes and a tendency to metastasize to different organs with lung, lymph nodes, liver, and bone being the most common sites of spread [2]. Recurrence rates are about 20-30% after radical nephrectomy for localized disease with less than 5% local and mostly distant metastases predominantly to the lung [1]. Metastases to the urinary tract from RCC are uncommon, especially in the absence of metastatic disease elsewhere in the body [2].

We report a rare case of metachronous residual ureteric stump metastasis eight years after the primary RCC was treated. The histology was chromophobe RCC in both types.

## Case presentation

**Figure 1:**
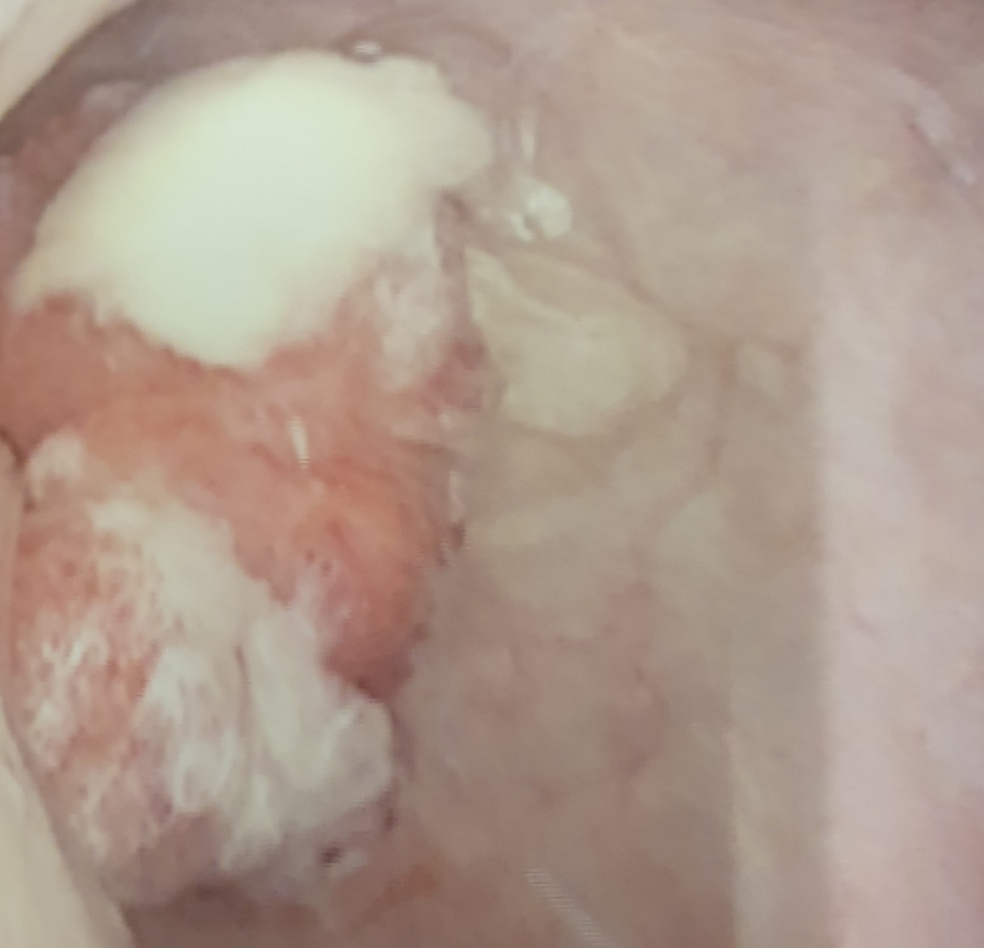
Cystoscopic view of the bladder tumor arising in the area of the right ureteric orifice

A 50-year-old female presented with visible hematuria in May 2007. Flexible cystoscopy was normal and an ultrasound of the upper tracts revealed a right renal mass suspicious of renal cancer. She underwent computed tomography (CT) of the abdomen, chest, and pelvis, which confirmed an extensive right renal tumor with no renal vein or caval thrombus. Bone scan did not show any evidence of bone metastases with radiologic staging as T2N0M0. She underwent right radical nephrectomy and the histology confirmed pathological T2 chromophobe renal cell carcinoma, which was excised completely and confined to the kidney. She was on regular follow-up surveillance and remained well. However, she presented in August 2011, four years later, with visible hematuria. She underwent investigations with a CT urogram and flexible cystoscopy, which were normal, and hence was discharged. She presented again in January 2014 with lower back pain, lower abdomen pain, and visible hematuria. A workup again was normal which included a contrast CT scan of the abdomen and flexible cystoscopy. In May 2015, she had recurrent episodes of visible hematuria. Flexible cystoscopy revealed a bladder tumor on the right lateral wall (Figure 1).

**Figure 2:**
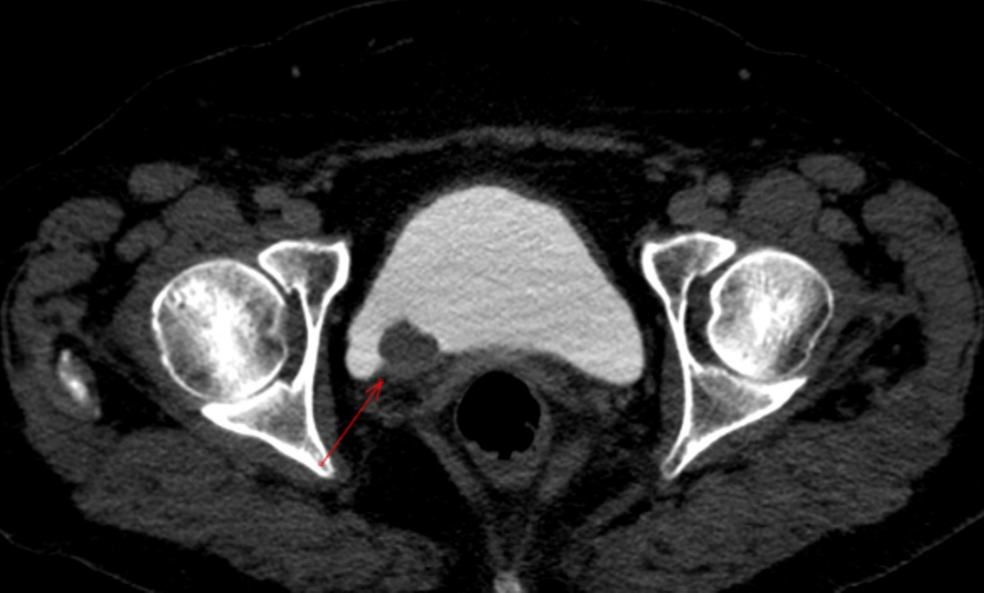
Axial computed tomography view of the tumor in the bladder (red arrow pointing to the tumor)

**Figure 3:**
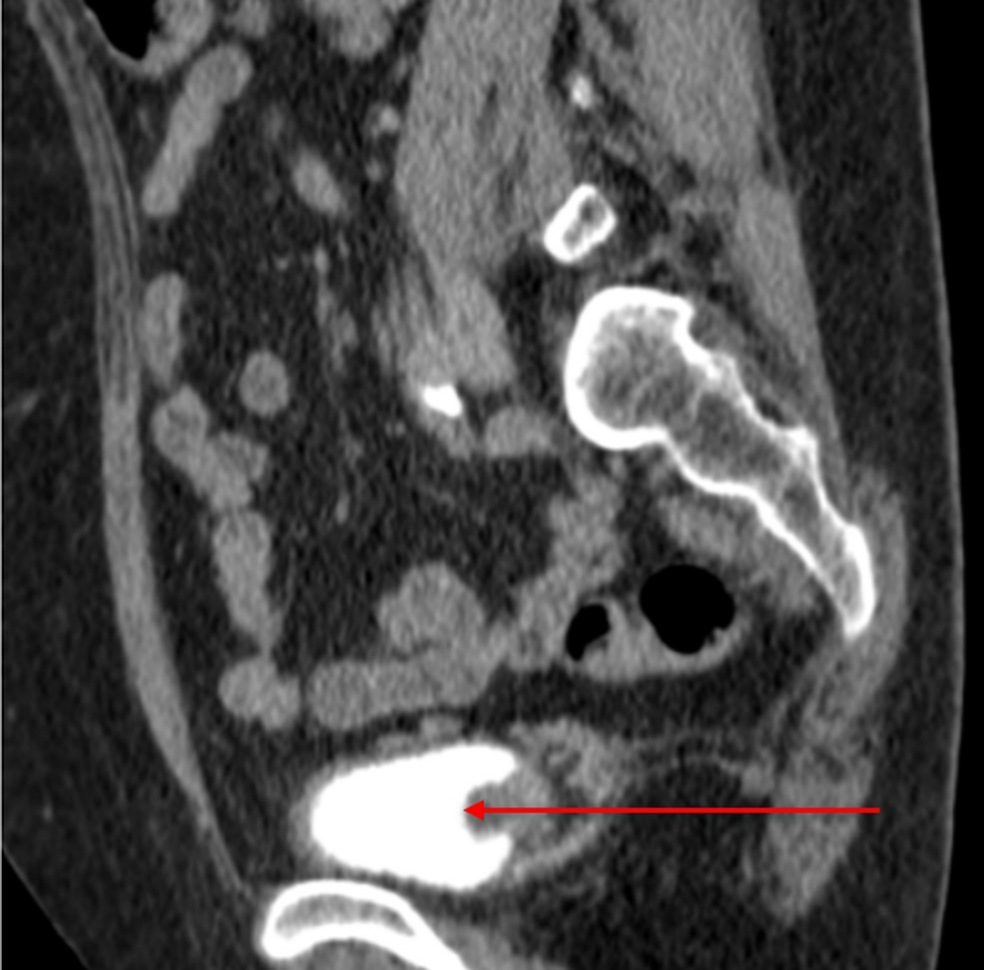
Coronal computed tomography view of the tumor in the bladder (red arrow pointing to the tumor)

A contrast CT scan of the abdomen, chest, and pelvis showed no metastatic lesion but did demonstrate a 2.1 cm polypoid lesion at the right vesicoureteric junction consistent with bladder tumor (Figures 2, 3).

**Figure 4:**
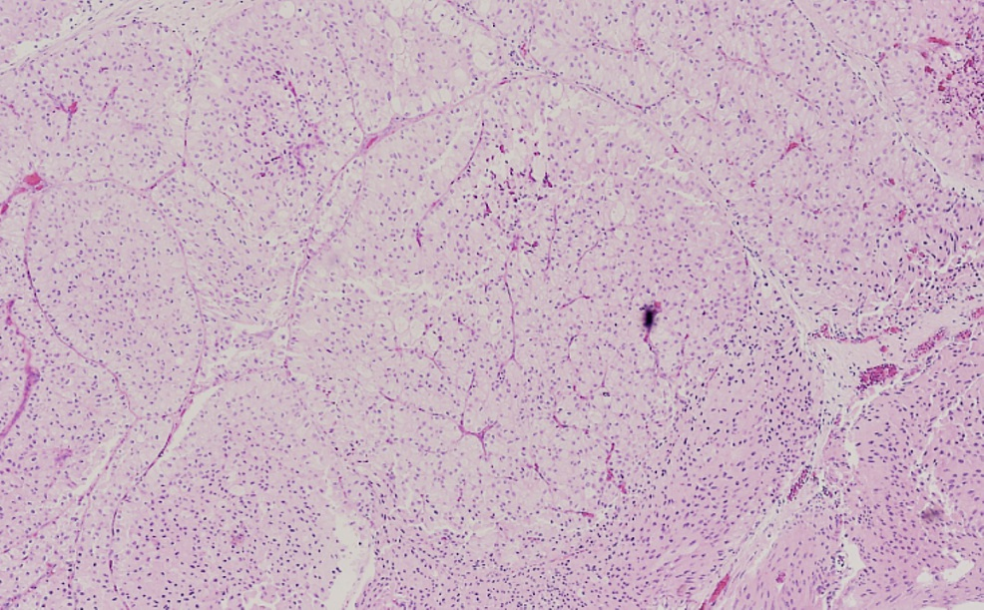
Low power view (hematoxylin and eosin) of the cut specimen of the tumor

**Figure 5:**
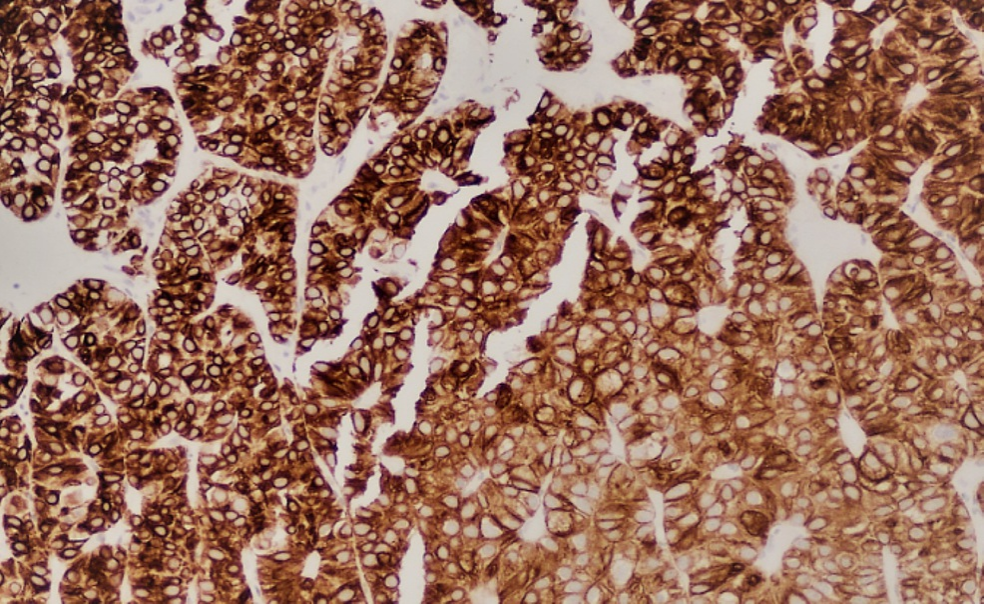
CK7 staining showing diffuse and strong expression of chromophobe renal cell carcinoma CK7, cytokeratin 7.

She underwent transurethral resection of the bladder tumor and the histology surprisingly came back as chromophobe renal cell carcinoma. At this stage, the histology from the nephrectomy specimen was reviewed again and this showed to be chromophobe renal cell carcinoma with no lymphovascular invasion (Figures 4, 5).

She underwent robotic-assisted right ureteric stump excision along with a cuff of the bladder in September 2015 and the specimen showed to be chromophobe renal cell carcinoma with negative margins. She remains well on the follow-up with no local or distant recurrence on surveillance of the bladder and CT of the chest and abdomen five years later.

## Discussion

RCC is classified into different subtypes based on the morphology and histopathologic features with clear cell carcinoma being the most common and accounting for 75%, papillary for about 10%, chromophobe for about 5%, and undifferentiated representing the remaining of the cancers [3]. Patients with chromophobe renal cell cancer tend to have an excellent prognosis as compared to the rest of the subtypes [1]. These tend to remain localized even when attaining considerable size and surgical resection offers a favorable long-term outlook [4].

Metastases to the urinary tract from renal parenchymal tumors are very rare and there are only a few cases reported in the literature, about 50 odd in number. The occurrence of this metastatic event has ranged from a few months up to eight years following radical nephrectomy [5]. The most common presenting feature is recurrent hematuria, which was seen in our case [6].

The very occurrence of this unusual and late metastasis from RCC brings to attention the mode of spread of RCC. It is quite usual to leave a ureteric stump behind following a standard radical nephrectomy and although ureteric stump metastases are rare, it is not unsusceptible [7].

Several theories have been postulated to explain this unusual pattern of metastases from RCC to the ureter and include blood-borne dissemination, lymphatic spread, and mucosal and submucosal seeding of the urothelium by the cancer cells, which reach the collecting system either by ulceration or direct spread [8]. The most plausible explanation for the isolated ureteric metastases could be the retrograde spread of the tumor cells from the renal vein into the ureteral vein, a theory which could be supported by the autopsy findings of bladder metastases in patients with renal vein involvement in a higher percentage (6.1%) as compared to lower risk of such metastases when the renal vein was not involved (1.2%) [6]. There has been a report of the retrograde venous spread of the RCC to the vagina, which could further support the above theory [9]. It is difficult to ascertain the exact cause of spread in our patient but any of the above factors could have contributed.

Our patient had recurrent presentations with visible hematuria lasting for four years before a tumor was seen in the bladder at the site of the ipsilateral ureteric opening. Though the patient was investigated with the recommended guidelines in place with cystoscopy and upper tract imaging for detection of urothelial cancer, no apparent cause was identified [10]. This would be consistent with the fact that the ureteric stump does not contribute to the urinary function any longer and any lesion in the stump may not be easily detected. Nonetheless, a CT scan would identify such abnormality, which was always performed in our case in all presenting episodes. However, it would seem pertinent to mention that diagnostic ureteroscopy was not considered for evaluation of the ureteric stump, which can prove challenging in such circumstances with difficult or impossible to catheterize the ureteric orifice. There could have been an argument to perform magnetic resonance imaging (MRI) when the patient had recurrent episodes of hematuria with no abnormality detected on conventional imaging and procedures. MRI has superior soft-tissue contrast as compared to CT scan, although CT has a better spatial resolution [11]. Although extremely rare, it is still possible to encounter a primary urothelial tumor of the ureteric stump after radical nephrectomy [12].

Ever since the last operation of the excision of the ureteric stump along with a cuff of the bladder, the patient remains on strict surveillance and to date, five years later, she is free of local and distant cancer recurrence.

## Conclusions

The above case highlights that metastases can develop any time after successful treatment of the primary RCC and this can be delayed beyond a standard surveillance program. Any visible hematuria in such patients demands thorough investigation and a ureteric stump tumor should be kept in mind and appropriately investigated for it including the recommended protocol and ureteroscopy if required in cases when no apparent abnormality is detected. Surgical excision along with a cuff of the bladder remains the standard treatment with a good prognosis in such cases in the absence of metastases elsewhere.

## References

[1] Motzer RJ, Bander NH, Nanus DM (1996). Renal-cell carcinoma. N Engl J Med.

[2] Gelister JS, Falzon M, Crawford R, Chapple CR, Hendry WF (1992). Urinary tract metastasis from renal carcinoma. Br J Urol.

[3] Vera-Badillo FE, Conde E, Duran I (2012). Chromophobe renal cell carcinoma: a review of an uncommon entity. Int J Urol.

[4] Macleod R, Kheirandish P, Ondego C, Biyani CS (2015). Chromophobe renal cell carcinoma recurrence in the ureter: a late presentation of a rare metastasis. Can Urol Assoc J.

[5] Oserowsky A, Allison D, Weinstein S, Nguyen V, Murray KS (2020). Metastasis of renal cell carcinoma to the distal ureteral stump beyond recommended baseline surveillance duration. Urol Case Rep.

[6] Cheng K-C, Cho C-L, Chau LH, Lam K-M, So H-S (2013). Solitary metachronous metastases of renal cell carcinoma to the ureter. Int J Case Rep Med.

[7] Psihramis KE (1987). Ureteral stump metastases from renal adenocarcinoma. Urology.

[8] Bobby VS, Kurien S, Gopalakrishnan G, Kekre NS (2002). Asynchronous ureteral stump metastases from papillary renal adenocarcinoma. Ind J Urol.

[9] Mitchell JE (1958). Ureteric secondaries from a hypernephroma. Br J Surg.

[10] Anderson MJ, Fawcett MD (2008). BAUS/RA Guidelines. Joint consensus statement on the initial assessment of
haematuria. Feehally J, Goldberg L, Kelly MJ, MacTier R, et al. Joint consensus statement on the initial assessment of haematuria. BAUS/RA Guidelines.

[11] Jaffe J, Friedman AC, Seidmon EJ, Radecki PD, Lev-Toaff AS, Caroline DF (1987). Diagnosis of ureteral stump transitional cell carcinoma by CT and MR imaging. AJR Am J Roentgenol.

[12] Masago T, Naka T, Yoshida H, Takahashi C (2019). Primary tumor of the ureteral stump after a radical nephrectomy for renal cell carcinoma: case report and literature review. Int Cancer Conf J.

